# A Rare Case of Frontal Lobe Cavernous Malformation Mimicking Meningioma

**DOI:** 10.7759/cureus.27027

**Published:** 2022-07-19

**Authors:** Mudit K Kumar, Varsha Rangankar, Aastha Agarwal

**Affiliations:** 1 Radiology, Dr. D. Y. Patil Medical College, Hospital & Research Centre, Pune, IND; 2 Radiodiagnosis, Dr. D. Y. Patil Medical College, Hospital & Research Centre, Pune, IND

**Keywords:** magnetic resonance imaging, computed tomography (ct), venous malformation, cavernoma, frontal meningioma

## Abstract

Cavernous venous malformations are benign vascular lesions that commonly occur in the brain parenchyma. These when present in the extra-axial or superficial cortical location can be mistaken for meningioma. Magnetic resonance imaging (MRI) can help in the detection and easy differentiation of the two entities and thus aid in preoperative diagnosis and preventing intraoperative complications. We present a case of an 18-year-old male patient suffering from seizures, which was initially diagnosed as meningioma. However, detailed evaluation with MRI raised a possibility of cavernous malformation and it was considered as a differential.

## Introduction

Cavernous venous malformations, also known as cavernomas, are benign vascular lesions that commonly occur in the brain parenchyma [1]. These are well-circumscribed, larger, clustered sinusoidal vessels that are isolated from parenchymal tissue by a single layer of the epithelium [2]. The sinusoidal vascular channels are found within the brain and are separated by fibrous tissue; nevertheless, there is no intervening neural parenchymal or major feeding or draining arteries or veins [3]. They are most commonly seen in the parenchyma of the brain, although they can also be found in the spinal cord and extra-axial sites [1].

In the general population, 5-13% incidence of cavernoma is seen in all vascular malformations in central nervous systems. Among the locations in the brain, supra-tentorial is the most common location [1]. Extra-axial cavernomas account for about 14% of all cavernomas [4].

Meningiomas are extra-axial common benign tumors originating from the meninges. Based on incidence, meningiomas are more common than cavernomas. Characteristic signs are calcifications and adjacent meningeal enhancement, also known as dural tail sign. Evaluation with computed tomography alone is difficult; magnetic resonance imaging (MRI) can help in better differentiation and diagnosis.

In this case report, a CT scan presented a diagnostic conundrum between a cavernoma and a meningioma. The use of MRI aided in the accurate identification and differentiation of cavernoma, which upon histological examination revealed to be a highly vascular cavernoma.

## Case presentation

An 18-year-old male patient presented to our hospital with an episode of seizure followed by loss of consciousness. He was suffering from recurrent episodes of generalized tonic-clonic seizures for the last 3 years and was on antiepileptic medication. There was no associated history of headache, vomiting, trauma, or past surgeries. The patient had no comorbidities and no history of addiction or drug abuse. The patient had undergone MRI and CT scan 6 months prior to coming to our hospital which showed an extra-axial lesion in the right parasagittal and parafalcine region along the anterior right frontal lobe with evidence of multiple calcifications with associated minimal scalloping of the frontal bone. The lesion was thought to be a meningioma.

**Figure 1:**
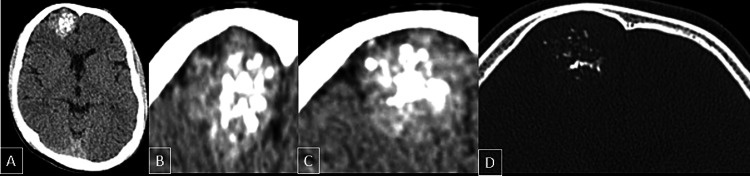
Non-contrast CT scan. The scan showed a cortical lesion in the anterior right frontal lobe in the right parasagittal plane with multiple hyperdense foci within (A-C), representing calcifications/low-grade hemorrhage.  Bone window reconstruction reveals bone scalloping and thinning of the right frontal bone overlying the lesion (D).

The patient came to our department for a follow-up CT brain (Figure 1) which showed a large well-defined cortex-based lesion in the parafalcine right anterior frontal region involving the cortical and subcortical regions showing mixed attenuation with multiple small variable sizes calcific and hemorrhagic foci within. The lesion was causing scalloping and thinning of the adjacent right frontal bone. We thought the lesion was cortical rather than extra-axial in location as was reported in the previous scan.

**Figure 2:**
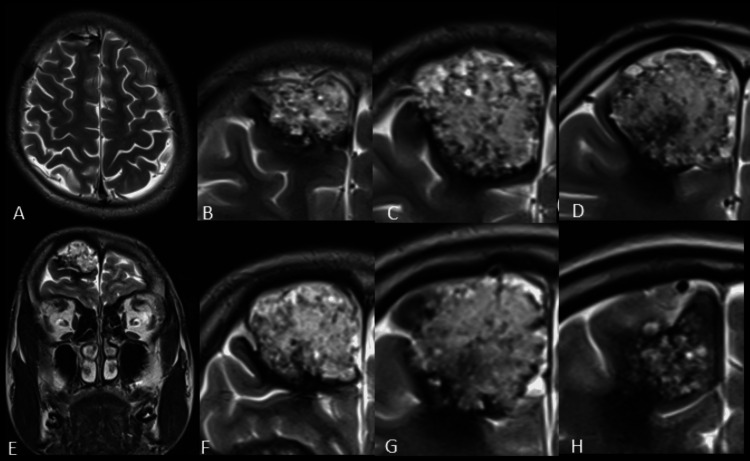
T2 weight axial (A-D) and coronal (E-H) MRI images. Images showed a well-defined heterogeneously hyperintense cortex-based lesion in the anterior frontal lobe in the right parasagittal plane.

**Figure 3:**
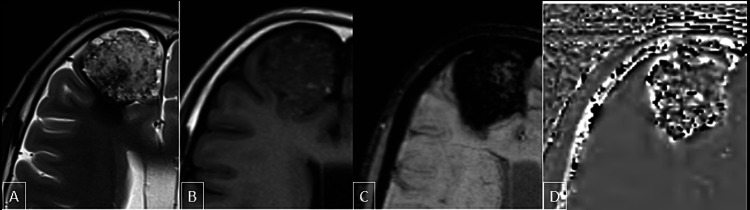
MRI brain scan. The scan showed a large well-defined cortex-based lesion in the anterior right frontal lobe in the right parasagittal plane which was heterogeneously hyperintense on T2 images (A), iso to hypointense on T1WI (B). Most of the lesions were showing blooming on SWI (C), some of the regions appearing dark and some bright on phase filtered images (D) suggestive of the presence of blood products and calcification within the lesion. The lesion also showed surrounding T2 (A) hypointense rim which is blooming on SWI (D) and appears bright on phase image suggestive of hemosiderin deposition. T1WI: T1-weighted image; SWI: susceptibility-weighted imaging

MRI brain revealed a large well-defined cortex-based lesion in the parafalcine right anterior frontal region; measuring about 32×31×30 mm (CC × TR × AP) (Figures 2-3).

**Figure 4:**
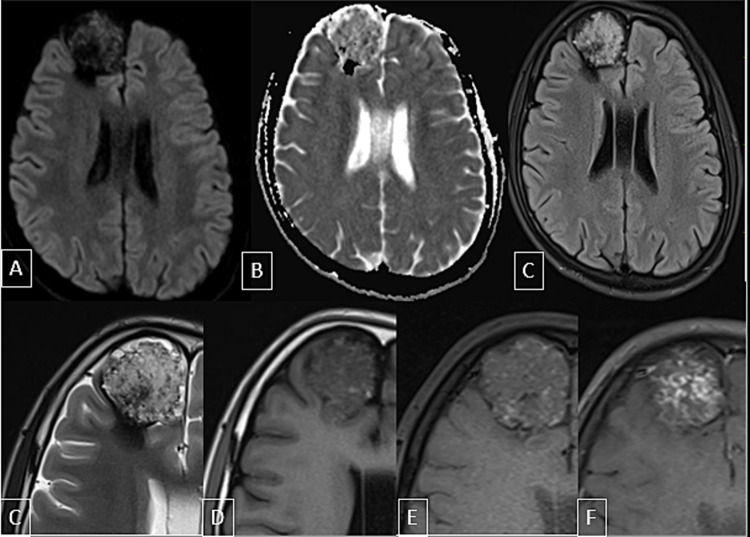
MRI brain scan. The scan showed a large well-defined cortex-based lesion in the anterior right frontal lobe in the right parasagittal plane showing no diffusion restriction (A-B). This lesion is appearing heterogeneously hyperintense on FLAIR (C) and T2-weighted images(D); iso to hypointense  on T1-weighted images (E). Multiple small T2 hypointense and T1 hyperintense foci are seen within the lesion (D, E). This lesion shows heterogeneous post-contrast enhancement on post-contrast T1FS images (F). FLAIR: fluid-attenuated inversion recovery; T1FS: T1-weighted fat-suppressed

The lesion was heterogeneously hyperintense on T2-weighted and fluid-attenuated inversion recovery (FLAIR) images, iso to hypointense on T1WI with no diffusion restriction. The lesion showed heterogeneous post-contrast enhancement on post-contrast enhancement fat-saturated T1-weighted images. Multiple small T1 hyperintense and T2 hypointense foci were seen in the lesion. Most of the lesions were showing blooming on susceptibility-weighted imaging (SWI) with some regions appearing dark and some bright on phase filtered images suggestive of the presence of blood products and calcification within the lesion. The lesion also had a surrounding T2 hypointense rim which was blooming on SWI and bright on phase images representing hemosiderin deposition (Figure 4). Few prominent arteries were seen adjoining the lesion with no hypertrophies feeding arteries or nidus identified.

**Figure 5:**
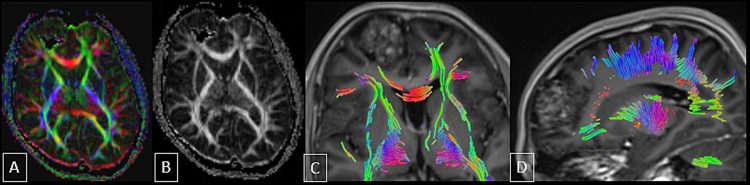
DTI and tractography images. The images showed disruption of the adjacent anterior portion of the right superior occipitofrontal fasciculus and arcuate fibers of the right frontal lobe in the region of the lesion. DTI: diffusion tensor imaging

Diffusion tensor imaging (DTI) and tractography (Figure 5) showed disruption of the adjacent anterior portion of the right superior occipitofrontal fasciculus and arcuate fibers of the right frontal lobe in the region of the lesion. The provisional diagnosis of cavernous venous malformation was made and meningioma was considered as a differential diagnosis.

**Figure 6:**
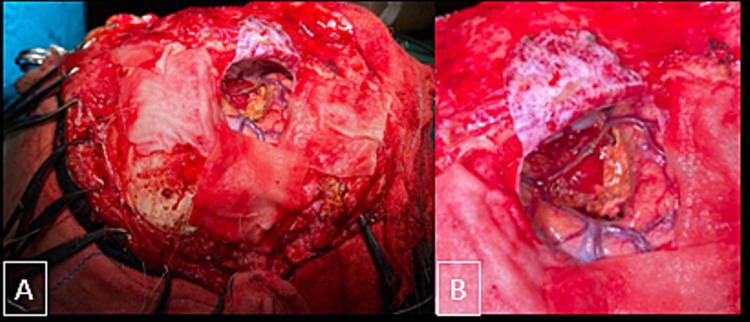
Intraoperative images. The images showed an intra-axial lesion giving typical mulberry appearance in the right parasagittal portion of the right frontal lobe adjacent to the bridging cortical veins. On histopathological analysis, cavernous malformation was confirmed.

The patient was subsequently operated on (Figure 6) and the histopathology specimen showed cavernous venous malformation with areas of hemorrhage, calcification, and reactive glial tissue.

## Discussion

Cavernomas have an unknown origin, however, cavernomas have been linked to cranial radiation, coexisting vascular malformation, genetic, and hormonal variables. In the hereditary type of cavernoma, the proportion of individuals who develop clinical symptoms is greater than in the sporadic variant [5].

The majority of the cases are asymptomatic and are incidentally detected on autopsy. Common symptoms are headache, hemorrhage, seizures, and focal or progressive neurologic deficits. Cavernomas cause mass effects and hemosiderin deposition which usually cause seizures [6]. New seizures are seen commonly in the supratentorial cavernomas and neurologic deficits are seen in the infratentorial cavernomas [4].

Cavernomas show a distinctive “mulberry” appearance with engorged purple clusters of vessels on the surface. The cavernomas are thin-walled dilated capillaries with a simple endothelial lining and a thin, fibrous adventitia on microscopic inspection. Muscle and elastic tissue are absent within the vessel walls. According to the classic description, no intervening brain tissue in between the vascular channels is seen [7].

As compared to the other vascular diseases, diagnosis of cavernous malformation is difficult since these are angiographically occult malformations [2]. MRI is considered an investigation of choice and helps in the accurate diagnosis.

Because it includes blood products of varying ages, a cerebral cavernous malformation shows a classic, well-defined lesion with popcorn-like, a mulberry pattern of varying signal intensities on T1- and T2-weighted imaging [7,8]. For parenchymal cavernous angiomas, a reticulated core with a hemosiderin ring and mixed-signal intensity is considered a diagnostic finding [9]. For identifying microhemorrhages and hemosiderin rings, SWI is more sensitive than MRI [1].

Even though the lesions are profoundly situated in eloquent regions, DTI is employed intraoperatively to better assess the lesions and surrounding tissue to improve the surgical result. The surgeon can see the white matter tracts that commonly pass over the hemosiderin rim of the cavernous malformation lesion using DT tractography [2].

Reticulated core and low-signal margin of hemosiderin are absent in dural cavernous malformations. Additionally, these are indistinguishable from meningioma and show intense and homogenous post-contrast enhancement [9]. The dural cavernous malformations also show dural tail signs, making the diagnosis difficult [4].

In contrast-enhanced CT, meningiomas appear homogeneous or heterogeneous, with a dural tail sign on MRI following gadolinium administration. Furthermore, even though angiographic tests are frequently negative, a small vascular redness may occasionally be observed. Even the most expert radiologists find it difficult to discern between the two before surgery [1]. In the present case, even though scalloping of the frontal bone was present, lack of hyperostosis favored the diagnosis of cavernous malformation than meningioma.

The main treatment is surgical resection. Because middle cranial fossa cavernous angiomas bleed abundantly, preoperative radiation and embolization have been proven to decrease blood loss after surgical excision. Outside the middle cerebral fossa, however, neither embolization nor radiation treatment is necessary for the effective removal of cavernous angiomas [9]. Complete excision was accomplished in our patient with minimum bleeding.

## Conclusions

Cavernous venous malformations in the extra-axial or superficial cortical location are often mistaken for meningioma. In comparison to classic cavernomas, the dural cavernous malformations and cavernomas along with the cerebral convexity lack reticular core and hemosiderin rings, show homogeneous intense enhancement and dural tail sign further adding to the diagnostic dilemma. It is very important to make the preoperative diagnosis of cavernous malformation because complete excise of the lesion without breaching the tumor capsule is essential to mitigate the risk of bleeding. MRI can help to differentiate cavernomas from meningiomas in such difficult cases.
